# Effect of solution pH on root architecture of four apple rootstocks grown in an aeroponics nutrient misting system

**DOI:** 10.3389/fpls.2024.1351679

**Published:** 2024-06-10

**Authors:** Ali Al Farqani, Lailiang Cheng, Terence L. Robinson, Gennaro Fazio

**Affiliations:** ^1^Horticulture Section, School of Integrative Plant Sciences, Cornell AgriTech, Cornell University, Geneva, NY, United States; ^2^Horticulture Section, School of Integrative Plant Sciences, Cornell University, Ithaca, NY, United States; ^3^U.S. Department of Agriculture, Agricultural Research Service (USDA ARS) Plant Genetic Resources Unit, Cornell AgriTech, Geneva, NY, United States

**Keywords:** apple rootstock, nutrient absorption, aeroponics, root architecture analysis, root growth

## Abstract

The pH of the solution in the rhizosphere is an important factor that determines the availability and mobility of nutrients for plant uptake. Solution pH may also affect the root distribution and architecture of apple rootstocks. In this study, we evaluated the effect of solution pH on root system development of apple rootstocks using an aeroponics system designed and developed at Cornell AgriTech Geneva, USA. Four Geneva^®^ apple rootstocks (G.210, G.214, G.41, and G.890) were grown in an aeroponic system under nutrient solution misting featuring continuously adjusted pH levels to three pH treatments (5.5, 6.5, and 8.0). Root development was monitored for 30 days and evaluated regularly for distribution and root mass. Images of the developed roots grown in the aeroponic system were collected at the end of the experiment using a high-resolution camera and analyzed using GiA Roots^®^ software, which generates root architecture parameter values in a semi-automated fashion. The resulting root architecture analysis showed that the Geneva^®^ rootstocks were significantly different for two architecture parameters. The length-to-width ratio analysis represented by two GiA Roots parameters (minor-to-major ellipse ratio and network width-to-depth ratio) showed that G.210 was flatter than G.890, which had a greater tendency to grow downward. Rootstocks G.214 and G.41 displayed similar growth values. The solution pH affected most root architecture parameter measurements where overall root growth was higher at pH 8 than at pH 5.5 and 6.5, which showed similar growth. In general, the average root width tended to decrease at higher pH values. While there were no significant differences in the leaf nutrient concentrations of P, K, Ca, Mg, S, B, Zn, Cu, and Fe within the four rootstocks, the pH level of the solution had a significant effect on P, Ca, and Mn. This study is the first of its kind to investigate the effect of pH on root architecture in a soil-free (aeroponic) environment and may have implications for apple root behavior under field conditions where pH levels are different.

## 1 Introduction

The root (stock) system and its architecture play vital roles in supporting overall fruit tree growth and productivity. In addition to their basic role in supporting the anchorage and storage of trees, root architecture and distribution through the soil profile influence the ability of the whole plant to acquire essential resources, such as water and nutrients (Lynch et al., 2014; Postma et al., 2014). As such, an increased understanding of the aboveground attributes and behavior of fruit trees should be accompanied by an increased understanding of the belowground properties in order to assess the whole tree characteristics that drive the overall growth and productivity of orchards. This is especially true for apple trees and other grafted production systems with two genotypes (rootstock and scion) grafted together. In recent years, our understanding of what drives the resilience, productivity, and fruit quality of apple scions has been attributed to specific rootstock qualities (Fazio et al., 2017; Lordan et al., 2019; de Macedo et al., 2021; Biasuz and Kalcsits, 2022; Fazio and Robinson, 2022). The genetic properties of apple rootstocks are associated with differential leaf and fruit mineral nutrient concentrations (Fazio et al., 2013; Fazio et al., 2020), hormone concentrations (Lordan et al., 2017), gene expression (Jensen et al., 2012), tree growth, and productivity (Autio et al., 2020).

Genotypic variance in root architecture and root growth dynamics has previously been described in apples (Fazio and Volder, 2009; Atucha et al., 2013; Mao et al., 2021), where fine roots are considered to be the most active and dynamic part of the root system (Emmett et al., 2014). They play an important role in scouting and navigating the soil environment for water and nutrients as well as nutrient uptake (Artacho and Bonomelli, 2016). The formation of lateral roots increases the sink strength of the root system and promotes the development of greater root length and soil penetration (Smet et al., 2012). This lateral exploration may ultimately lead to better nutrient and water acquisition (Zhu and Lynch, 2004), although some studies have suggested that lateral root formation may be detrimental to the absorption of nitrogen (Lynch, 2013; Postma et al., 2014; Zhan et al., 2015).

Apple root systems have distinctive seasonal growth patterns that influence nutrient and water exploration, along with the formation of symbiotic colonization with mycorrhizae (Eissenstat et al., 2006). Apple root growth has traditionally been described using rhizotron systems (Atkinson, 1989; Wells and Eissenstat, 2001; Yao et al., 2006; Ma et al., 2013; Fazio, 2022) and conventional shovelomics (Tracy et al., 2015). Considering the difficulty accessing underground environment they are associated with, these approaches have their benefits, but suffer from difficulties connected with limited spatial sampling and intensive image analysis needs for rhizotrons and in the case of shovelomics the destructive nature of sampling. An alternative that has been investigated by the apple rootstock breeding program in Geneva, NY, is the use of aeroponics to study the root development and architecture of newly released rootstocks (Fazio, 2017). Aeroponic growing systems have been used in multiple studies to investigate root development in several plant species but have been limited to small plants and shrubs (Jie and Kong, 1998; Christie and Nichols, 2003; Gregory et al., 2009). Different configurations of tanks, spray, temperature, and nutrient regimens can be used to study different root-related topics (Aidoo et al., 2019; Chowdhury et al., 2020; Cai et al., 2023; Min et al., 2023). In the case of apple rootstocks, aeroponic systems allow easy access to roots for imaging, precise chemical control of the nutrient solution being misted on the roots, and easy access for sampling. In addition, root growth and distribution are primarily driven by genetic makeup and are not restricted by soil boundaries. Although this may be considered a hindrance, as some root characters are highlighted by their interactions with soil, it simplifies the understanding of some characteristics by eliminating media-specific characteristics. It also enables repetitive, non-destructive observations by image analysis with GiA-Roots (Galkovskyi et al., 2012), a software package that measures root growth parameters, such as root surface area, fine root diameter, branching, total convex area, and root network area.

One of the major environmental factors influencing plant growth is soil acidity/alkalinity (pH), and its associated effects on nutrient availability, uptake, and bio-rhizosphere composition (Neilsen et al., 1982; Wang et al., 2012; Nurtaza et al., 2022). Varying pH levels in soil and/or irrigation water tend to modify the mobility and availability of nutrients, such that when they interact with different rootstock genotypes, they produce very different orchard growth and productivity patterns (Thomidis and Tsipouridis, 2005; Vrsic et al., 2016; Ghimire and Vashisth, 2019). Several studies have tested and confirmed the field performance of new apple rootstocks (Russo et al., 2007; Autio et al., 2011; Autio et al., 2020) and one preliminary study investigated the effect of pH on leaf nutrients (Fazio et al., 2012a); however, there is a paucity of information on the interaction of pH levels with new apple rootstocks. Aeroponic systems have been used successfully to investigate the effects of varying pH levels on the roots of other plant species (Svecová et al., 2023), resulting in a greater understanding of this interaction and development-related methodologies. One of the goals of this study was to develop and utilize aeroponic methodologies to monitor and evaluate the response of apple rootstocks grown *in vivo* to different solution pH values with repeated and accurate sampling and growth measurements as well as nutrient uptake.

## 2 Materials and methods

### 2.1 Aeroponic system design

**Figure 1:**
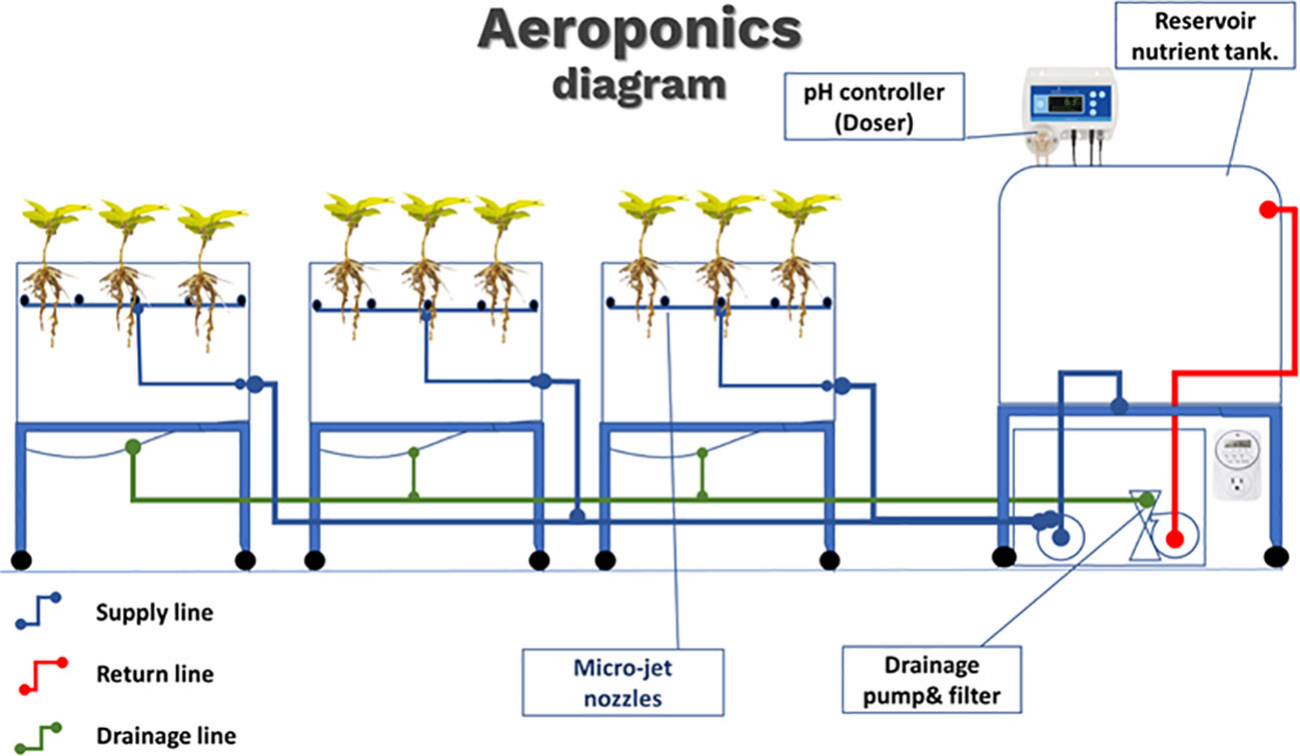
Diagram of aeroponic system. Three systems were deployed, one for each pH value (5.5, 6.5, and 8.5). A pH controller was used to maintain a constant pH of the nutrient solution. Delivery pump and nozzle system (blue) and drainage line (green) with return pump, line, and filter (red).

**Figure 2:**
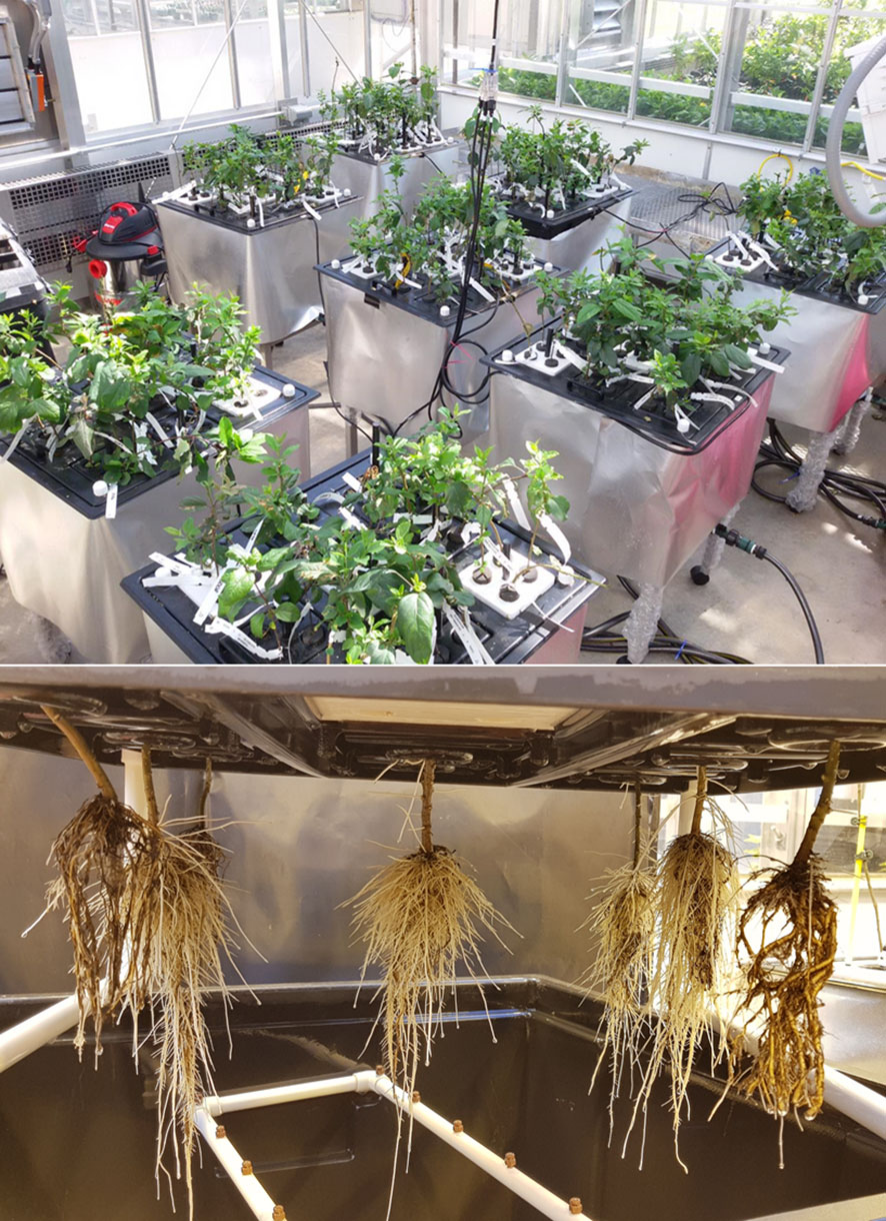
The aeroponic system was set up as plants grew actively. Three pH zones and rootstock plants were randomized within each zone. The plants were suspended in a misting chamber and received a periodic spray of a nutrient solution.

The aeroponic system used in this study followed the general guidelines used in previous studies (**Figures 1**, **2**) (Christie and Nichols, 2003; Min et al., 2023). High-density polyethylene (HDPE) black tanks covered with reflective film were used in this experiment. Nine tanks served as nutrient solution reservoirs to hold plants. Each tank was 60 cm × 60 cm × 50 cm (length × width × height) and was equipped with a top cover with nine square- shaped openings with square lids. A foam collar insert was placed at the center of each lid to hold the plant in an upright position, and the root system was suspended. The spacing between the centers of the collar insert and adjacent collar was 20 cm. Inside each tank, inner spraying rails positioned 25 cm under the top cover with 14 misting nozzles were installed to provide a fine mist of nutrient and oxygen mixture to the root system. The spraying rail was designed in a rectangular shape to ensure complete coverage of the nutrient mist surrounding the root system. Ten side misting nozzles had a 360° spray pattern, whereas the four corner nozzles had a 180° spray pattern. Side inlet and bottom drainage fittings were installed in each tank to maintain the nutrient circulation. The tanks were positioned on a mobile platform to facilitate their movement and accessibility.

Polyethylene resin tanks were used maintain a nutrient solution. The dimensions of the tanks were 30 cm × 30 cm × 90 cm (length × width × height). Two fittings were installed in the nutrient reservoir tanks to serve as feed and return lines at heights of 40 cm and 60 cm, respectively.

Two pumping systems were installed in the aeroponic system. A one-half horsepower rotary positive-displacement single-stage mechanical pump was used to supply nutrients from the nutrient reservoir to the aeroponics tanks. Another one-eighth horsepower magnetic-drive pump (Littlegiant. Franklin Electric Co., Inc., USA) was used to drain the aeroponic tank and return the nutrient solution to the reservoir. The nutrient solution was filtered twice, before passing through the pump and before entering the reservoir tank, to prevent particle blockage within the system. To control the misting at the required time interval and circulating the nutrients, digital timers were linked to the pumps and power supply.

The misting system was set to spray for 10 s every 120 s, with the circulating pump set to drain the nutrient solution for 90 s every 8 min. The aeroponic unit was kept in a greenhouse at temperatures of 25°C–30°C and a relative humidity of approximately 60%, while the temperature within the mist chamber (rhizospheric zone) was 25°C–30°C with 80%–90% of relative humidity.

### 2.2 Stock nutrient solution

Solution nutrients were prepared following the concentration recommended by the manufacturer of 1 g per 3.8 L of water using Jack’s nutrients 5-12-26 (JR PETERS Inc., USA). The solution was prepared by mixing nutrient fertilizers with warm reverse osmosis (RO) water, and the pH of the solution was adjusted to 5.5, 6.5, and 8.0. The acid formulation used food-grade phosphoric acid to lower the pH, whereas the base formulation used potassium hydroxide and potassium carbonate to increase the pH to the required level. The pH was continuously monitored and adjusted using a pH dosatronic system (Bluelab^®^ Corporation Limited, New Zealand). The volume of the nutrient solution in each nutrient reservoir was maintained at 90 L throughout the experiment. On a weekly basis, the nutrient solution was drained, and the system was flushed with filters replaced with fresh nutrient solution.

### 2.3 Plant material

The plants used in this experiment were 1-year-old tissue culture-propagated rootstocks, supplied by a commercial nursery (North American Plants Inc., McMinnville, OR, USA). Four commercially available apple rootstocks, Geneva^®^ 41 (G.41), Geneva^®^ 210 (G.210), Geneva^®^ 214 (G.214), and Geneva^®^ 890 (G.890) were selected for further investigation.

Prior to planting into the aeroponic system, plants were grown in pots with potting mix soil for one year. A preliminary assay was conducted to screen and evaluate individual plants based on their consistent height and stem diameter. When the stem diameter reached 5 mm–6 mm, the plants were removed from the potting soil, washed, and sprayed with fungicide according to the manufacturer’s instructions (Ridomil^®^, Syngenta-US.com). Plants were grown in an aeroponic system for 7 days with intact roots, and approximately 50% of the lower roots were pruned away using sterilized scissors to maintain uniform size, and all old and yellow leaves were removed. After three more days, all old roots were removed from all plants by cutting away the lower 1 cm–2 cm of the stem where the old roots were. Two plants from the same rootstock were randomly placed into each tank. Regular pest and disease inspection and application were carried out during the four weeks of growth. Ten days after plugging, plants were topped to a height of 35 cm.

### 2.4 Experimental design

Three aeroponic tanks were connected to a single nutrient reservoir using a flexible polyethylene hose, where the pH of the solution was monitored and regulated using a pH dosatronic (Bluelab^®^ Corporation Limited, New Zealand). Three solution pH treatments were used: 5.5, 6.5, and 8.0. Each treatment consisted of three aeroponic tanks distributed in rows. Uniform apple rootstocks were selected and plugged into designated aeroponic tanks (see **Supplementary Figures S1**, **S2**).

The experimental layout was a full factorial, consisting of four rootstocks (G.41, G.210, G.214, and G.890) and three pH treatments (pH 5, 6.5, and 8). Each pH treatment was replicated three times (cycles). Due to a space limitation with only three connected systems (one connected three-tank system attached to a nutrient solution reservoir; see **Supplementary Figure S1**) and the ability to test only three pH treatments at a time, the experiment was conducted in three cycles to replicate the pH treatment, ensuring that we assigned the pH treatment to the tank system/nutrient reservoir. In November 2018, the first set of 72 plants (three pH treatments, four rootstock genotypes, and six replicate plants per pH × genotype treatment) was plugged into the aeroponics to represent cycle one. After four weeks of growth, the plants were removed from the system, root growth was assessed and photographed, and the leaves were harvested for nutrient analysis. The second cycle was plugged in December 2018 with identical rootstocks and treatments, and harvested in January 2019. The third and final cycle was plugged in January 2019 and harvested in February 2019.

### 2.5 Image collection and processing

At the completion of each cycle, the plants were removed from the aeroponic tanks, and root images were taken for further image analysis and comparison. Each plant was held in an upright position to obtain a high-resolution image of the one-dimensional picture (see **Supplementary Figure 3**), with a metric ruler for dimension adjustment. All root images were taken from exactly the same position with a consistent distance to maintain the aspect ratio. Images were collected using a Canon EOS 50 D DSLR camera.

**Figure 3:**
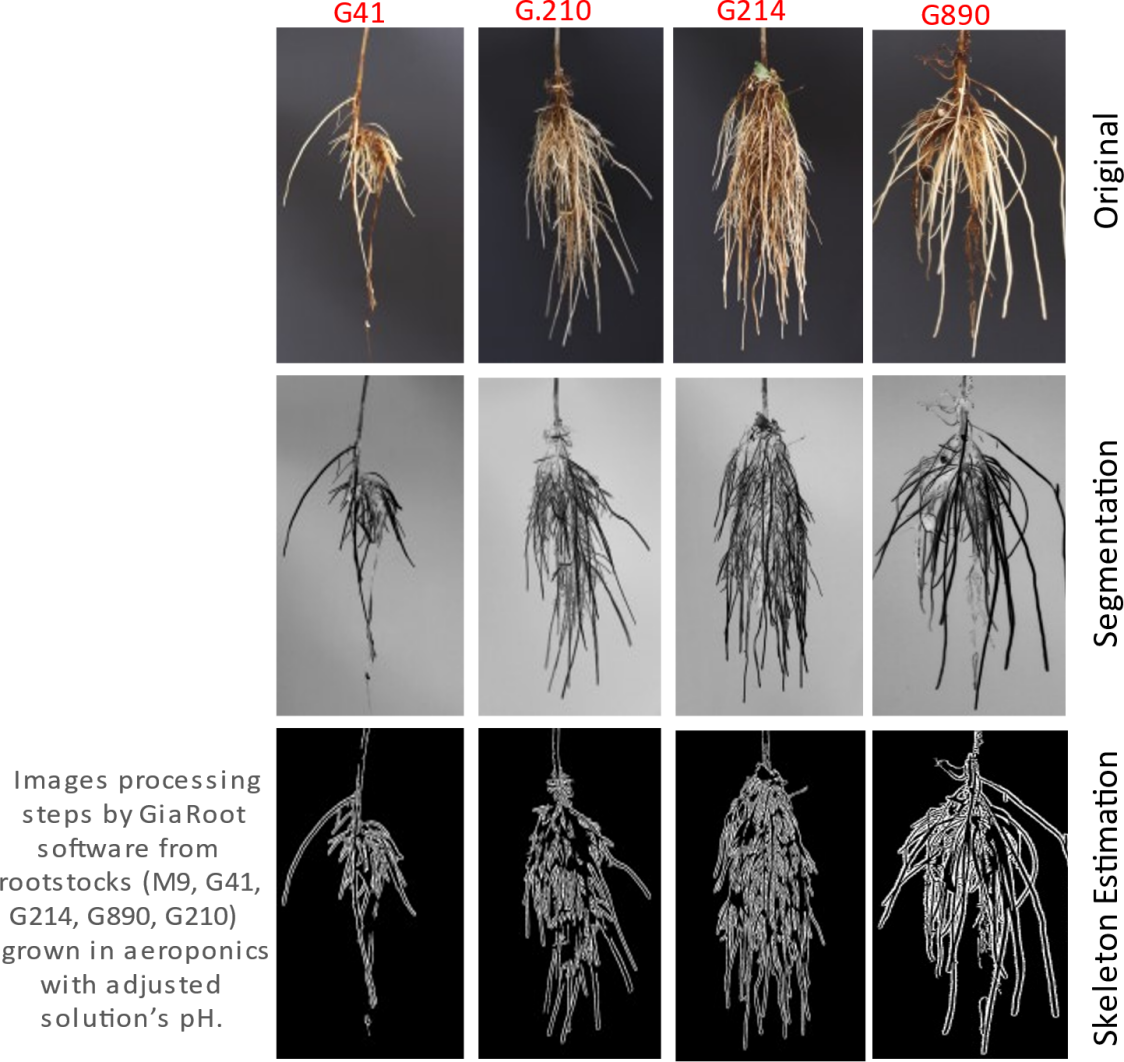
Example of root image processing steps in preparation for automated analysis and root architecture descriptor evaluation in GiA Roots. Four rootstock plant samples are shown.

All photos were processed using GiA Roots (General Image Analysis of Roots, Georgia Tech Research Corporation, and Duke University) (Galkovskyi et al., 2012). GiA Roots is a high-throughput software tool for automating and facilitating large-scale analysis of root systems, architecture, and networks (**Figure 3**). GiA Roots have been designed to help scientists and breeders quantify the structure of plant root system architecture, regardless of prior training in mathematics and computer science. Briefly, high-definition photos were processed as follows: (a) individual photos were calibrated, where measurements were converted from pixels to centimeters; (b) photographs were converted to grayscale according to software-recommended thresholds; (c) images were then processed by the double adaptive image thresholding algorithm to generate the output. The output parameters describing root architecture (AR), distribution (DI), dynamics (DY) and development (DE) were: Average Root Width (DE and DY), Network Bushiness (AR and DI), Ellipse Axes Ratio (DI), Major Ellipse Axis (DI), Maximum Number of Roots (DY and DE), Network Width (DI), Minor Ellipse Axis (DI and AR), Network Area (DI), Network Perimeter (AR and DI), Network Solidity (AR, DE and DY), Network Surface Area (AR and DI), Network Length (AR, DY and DI), Network Volume (DI and AR), Network Width to Depth Ratio (AR and DI), Network Length Distribution (AR and DI), Network Depth (AR and DI), Median Number of Roots (DY and DE), Network Convex Area (AR and DI) and Specific Root Length (DY and DE). For a detailed description, please refer to the **Appendix Supplementary Material** section and/or the original manuscript describing the software.

### 2.6 Leaf nutrient analysis

At the end of cycle 3, 10 leaves on new growth were collected from three trees per replicate for nutrient analysis. The leaf samples were washed three times with DI water and then dried at 70°C for 10 d. The samples were ground and placed in plastic-lined paper bags. All nutrient analyses were conducted at the Cornell Nutrient Analysis Laboratory (Ithaca, NY, USA) using inductively coupled plasma (ICP) atomic emission spectrometry.

### 2.7 Statistical analyses

**Figure 4:**
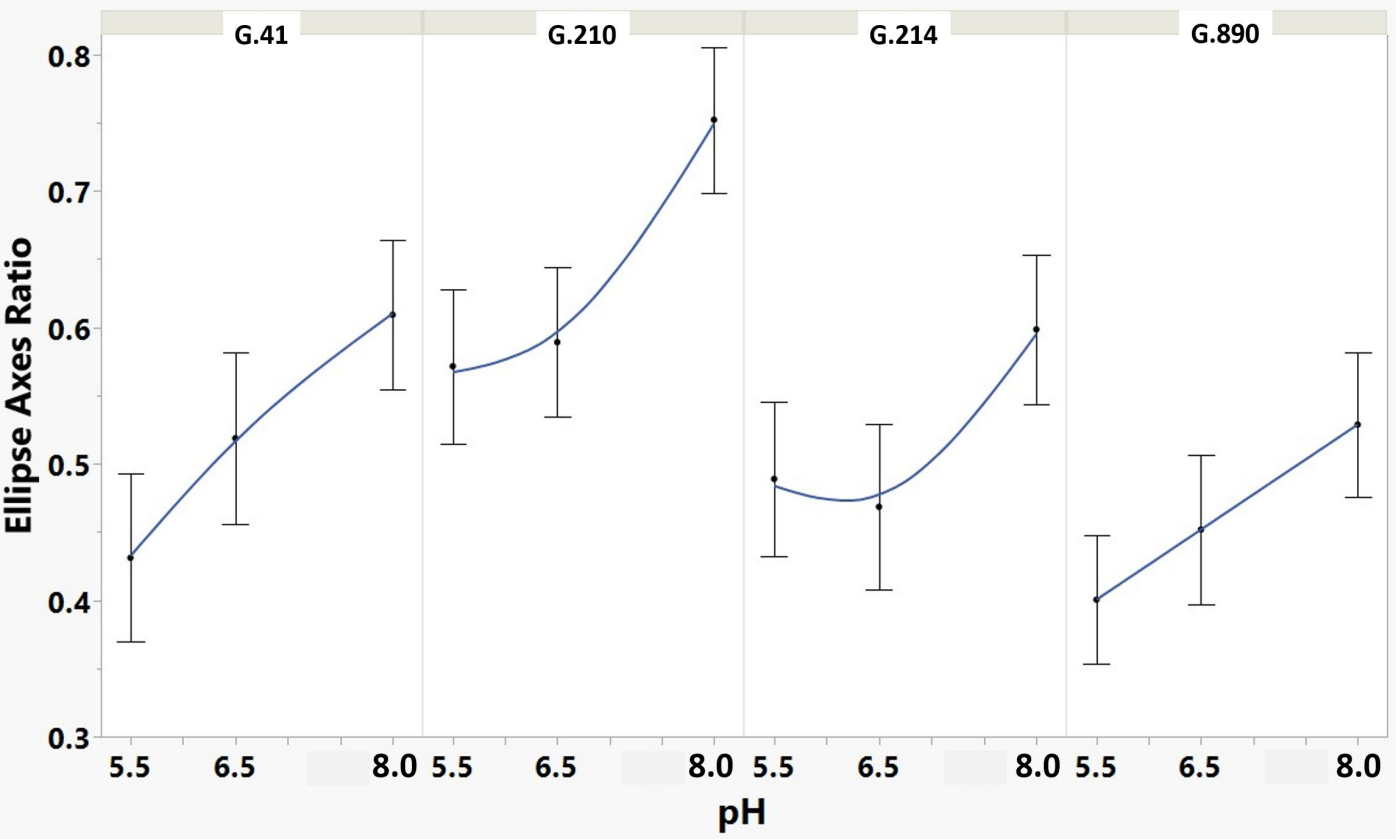
Means and SE for effect of rootstock genotype and solution pH on ellipse axis ratio root architecture descriptor of plants grown in aeroponic systems. After fitting an ellipse around the root system, this descriptor considers the ratio of the minor axis to the major axis. Equal axis lengths produce a circle (ratio of 1) and, therefore, describe a more rounded root system, whereas lower ratios describe more lengthened root systems.

**Figure 5:**
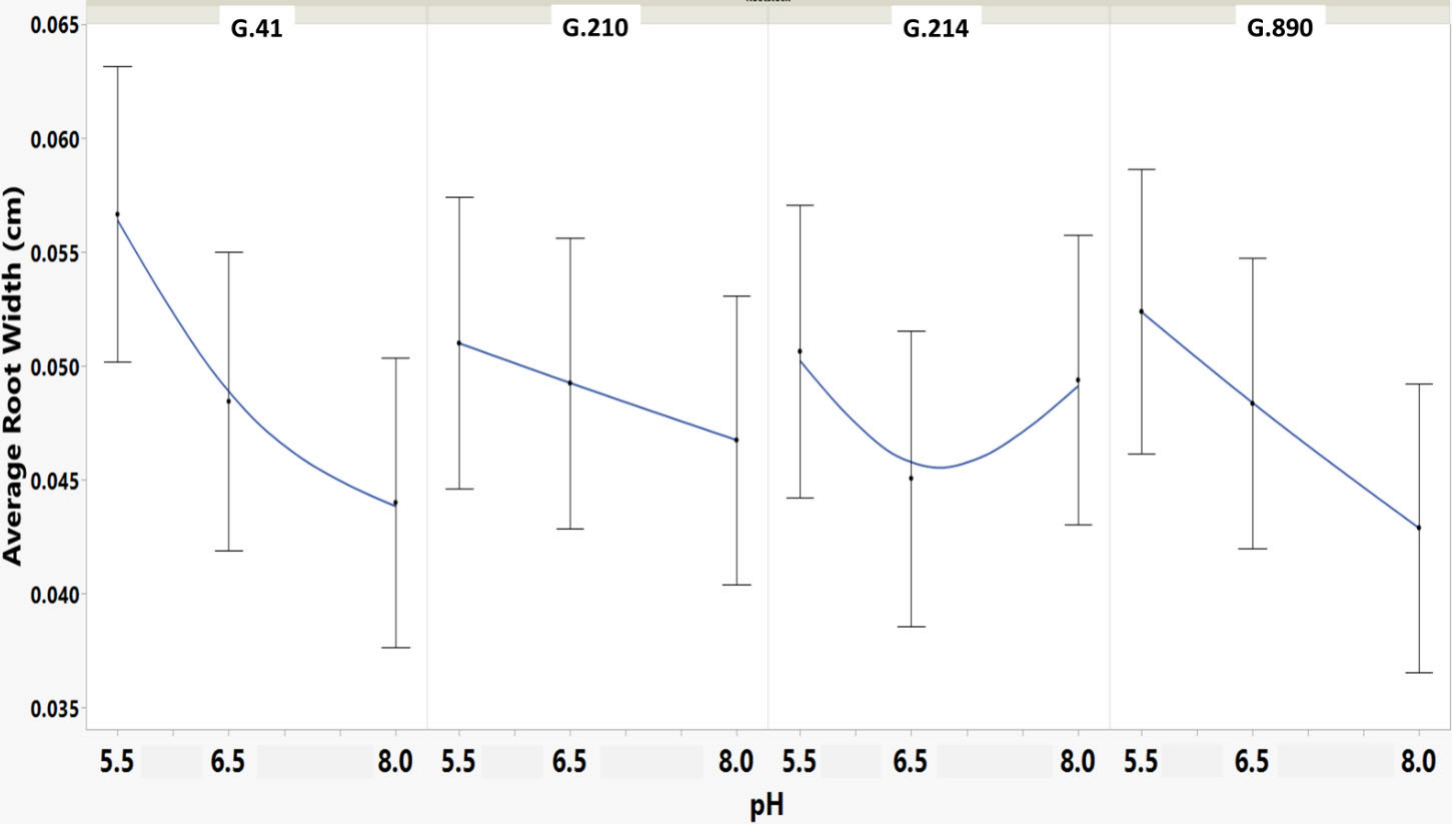
Means and SE for effect of rootstock genotype and solution pH on Average Root Width root architecture descriptor of plants grown in aeroponic systems. This descriptor shows the mean value of the root-width estimation computed for all pixels of the medial axis of the entire root system.

**Figure 6:**
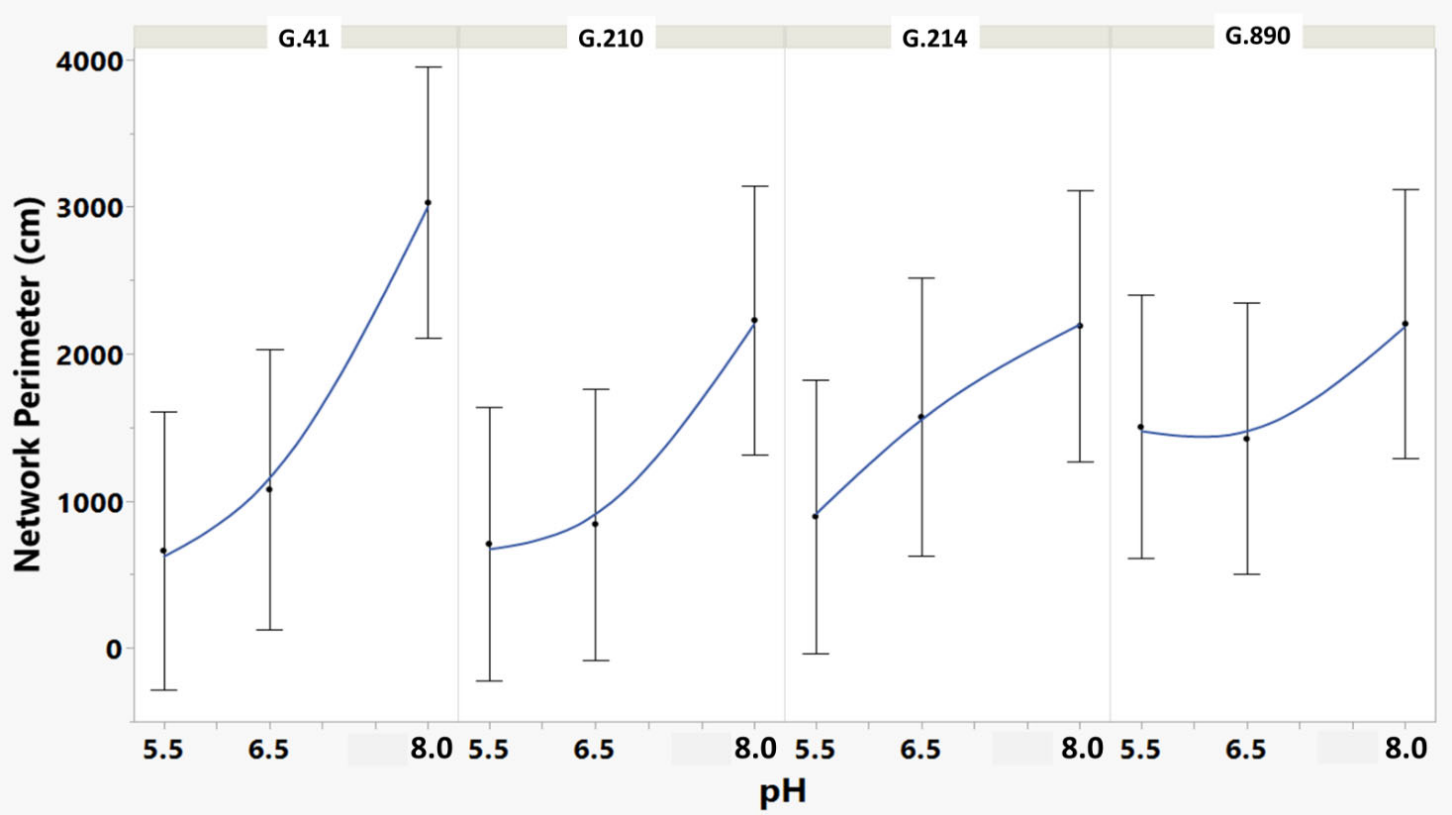
Means and SE for the effect of rootstock genotype and solution pH on Network Perimeter root architecture descriptor of plants grown in aeroponic systems. This descriptor is the total number of pixels connected to a background pixel (using an 8-nearest neighbor neighborhood) and then converted to centimeters. More roots produced a higher value for this descriptor.

Data analyses and graphics were performed using the SAS JMP 17 Pro statistical software package (SAS Institute Inc., Cary, NC, USA). The data from the three cycles were combined into a single dataset. The same data were subjected to multivariate analysis to generate Pearson correlation matrices and clustered correlation graphics for the entire dataset. Mixed Model Analysis with a full factorial for fixed effects (Rootstock, pH, Rootstock × pH interaction) and repeated measures (Cycles) was performed. Least square means were generated for the main effects and the interaction and subjected to the Tukey HSD procedure, separating means by contiguous letters (**Table 1**; **Supplementary Tables S1**, **S2**). A similar statistical treatment was performed on the output of the ICP nutrient analysis, and least square means were generated for the main effects and interactions. Interaction means (rootstock genotype × pH) of a selection of root architecture parameters combined with all nutrient parameters were used in the Hierarchical Clustering feature of JMP Pro 17 with dual clustering analysis (Ward method) to view the relationship between the architecture and nutrient datasets. Pearson correlation coefficients were generated for the relationships between P (%), K (%), Ca (%), Mg (%), S (%), B (ppm), Zn (ppm), Cu (ppm), Fe (ppm), Mn (ppm), Net. Convex Area (cm^2^), Net. Solidity (cm), and Net. Length Distr. (cm) for the overall data (all pH levels together) and separately for each pH treatment (**Supplementary Figures S4A–D**), and clustered by the level of correlation among the variables to ease the visualization of such relationships. Interaction plots for the Ellipse Axis Ratio, Average Root Width, and Network Perimeter (**Figures 4**–**6**) were generated using the Minitab^®^ 21 Statistical Software (www.minitab.com).

**Table 1:** Least square means and standard errors from mixed model analysis (main effects = pH), Tukey HSD and P-values for all GiA Roots root architecture variables.

Root Architecture Variable	pH5.5	Tukey HSD	pH 6.5	Tukey HSD	pH 8	Tukey HSD	Prob >F
*Average Root Width (cm)*	0.052	a	0.047	b	0.045	b	0.0001
*Std Error*	0.006		0.006		0.006		
*Network Bushiness*	1.696	a	1.613	a	1.678	a	0.4167
*Std Error*	0.094		0.096		0.093		
*Number of Connected Components*	2.766	b	6.195	b	12.695	a	<.0001
*Std Error*	3.494		3.518		3.480		
*Network Depth (cm)*	16.638	b	16.364	b	20.008	a	0.0047
*Std Error*	2.952		2.969		2.941		
*Ellipse Axes Ratio*	0.472	b	0.507	b	0.622	a	<.0001
*Std Error*	0.035		0.036		0.034		
*Network Length Distribution*	0.614	a	0.584	a	0.426	b	0.0003
*Std Error*	0.031		0.032		0.028		
*Major Ellipse Axis (cm)*	15.478	b	16.191	b	24.305	a	<.0001
*Std Error*	4.437		4.460		4.423		
*Maximum Number of Roots*	26.765	b	35.281	b	45.969	a	<.0001
*Std Error*	12.288		12.339		12.257		
*Network Width (cm)*	7.486	b	7.962	b	18.686	a	<.0001
*Std Error*	3.657		3.685		3.639		
*Median Number of Roots*	17.081	b	22.555	ab	29.148	a	0.0002
*Std Error*	9.161		9.192		9.142		
*Minor Ellipse Axis (cm)*	6.578	b	7.837	b	16.670	a	<.0001
*Std Error*	3.474		3.496		3.461		
*Network Area (cm2)*	24.778	b	25.260	b	47.503	a	<.0001
*Std Error*	14.669		14.712		14.643		
*Network Convex Area (cm2)*	82.385	b	104.659	b	474.5406	a	<.0001
*Std Error*	136.982		137.708		136.533		
*Network Perimeter (cm)*	941.366	b	1,228.089	b	2,412.225	a	<.0001
*Std Error*	863.513		866.347		861.773		
*Network Solidity*	0.252	a	0.233	a	0.177	b	<.0001
*Std Error*	0.013		0.014		0.012		
*Specific Root Length (cm)*	404.370	b	484.290	ab	536.128	a	0.0103
*Std Error*	118.150		118.701		117.810		
*Network Surface Area (cm2)*	101.060	b	103.540	b	196.981	a	<.0001
*Std Error*	61.378		61.561		61.266		
*Network Length (cm)*	563.103	b	771.641	b	1,547.550	a	<.0001
*Std Error*	561.257		563.169		560.082		
*Network Volume (cm3)*	1.994	b	1.717	b	3.169	a	<.0001
*Std Error*	0.861		0.864		0.859		
*Network Width to Depth Ratio*	0.501	b	0.522	b	0.805	a	<.0001
*Std Error*	0.056		0.058		0.055		

Letters a and b represent two significantly different statistical groups according to the Tukey Honestly Significant Difference (HSD).

## 3 Results

### 3.1 Aeroponic system

While the aeroponics system had been used in previous experiments featuring the root architecture of segregating populations (Fazio, 2017), this was the first time that we used the systems to measure the root’s responses to different pH values. Overall, the systems performed well, requiring constant maintenance and monitoring as different components (mostly pumps), which relied on the electrical grid, appeared to be the weakest link in the system. This system was built using flexible hoses for the delivery and return of the solution system. While maintaining the plants (walking around the systems) in cycle 1, some flexible hose connections were knocked loose but were reconnected in a timely manner, which was remediated by visible signals and adhesive tape. In a newer version of the system after this experiment was completed, we opted for PVC tubing and glue to avoid this problem. The pH controlling systems that were used to maintain a certain pH in the supply tanks were monitored daily, except for the pH 5.5 treatment in cycle 1 that took the pH to 5 for the duration of that cycle, was performed as described by the manufacturer. Other aeroponic designs include an aeration system that aids in the oxygenation of the solution; however, because this was a recirculating system, the solution had the chance to be aerated during the return discharge into the 90 L reservoir tank, foregoing the need for an additional mixing system.

### 3.2 Root growth

**Figure 7:**
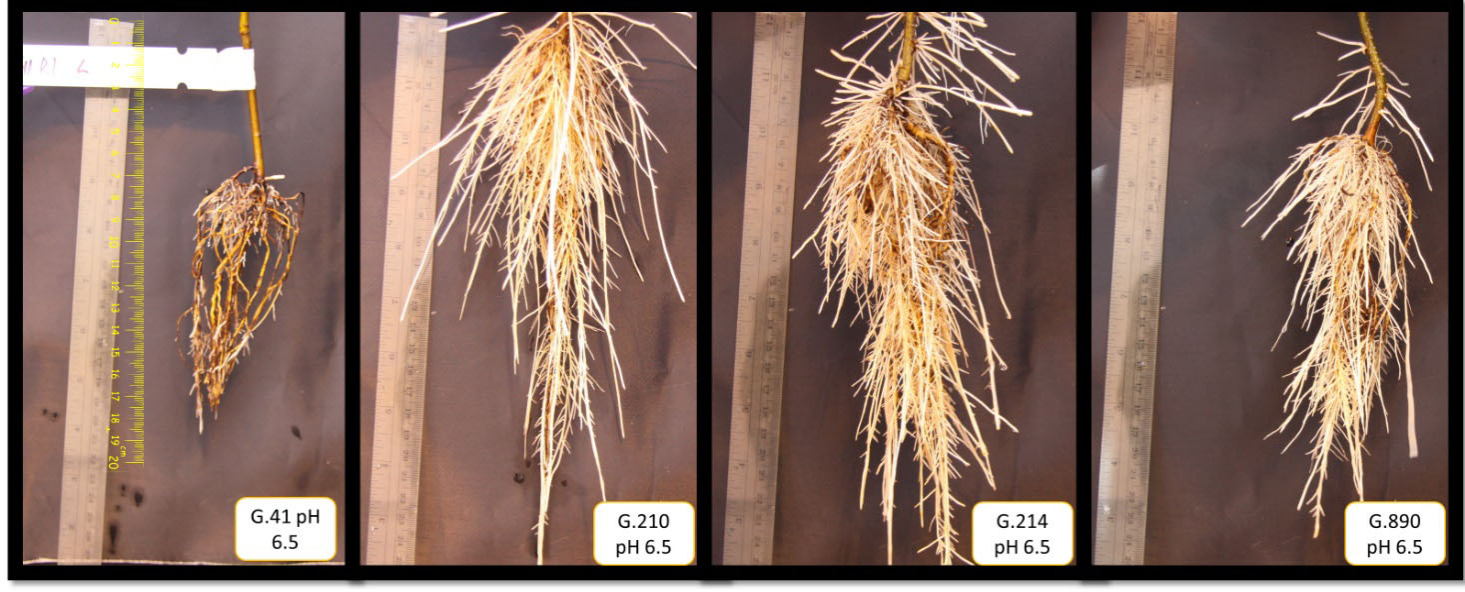
Side-by-side comparison of sample root systems representing each rootstock genotype (from left to right: G.41, G.210, G.214, and G.890) grown at pH 6.5 during cycle 2. The images were adjusted to a height of 25 cm.

**Figure 8:**
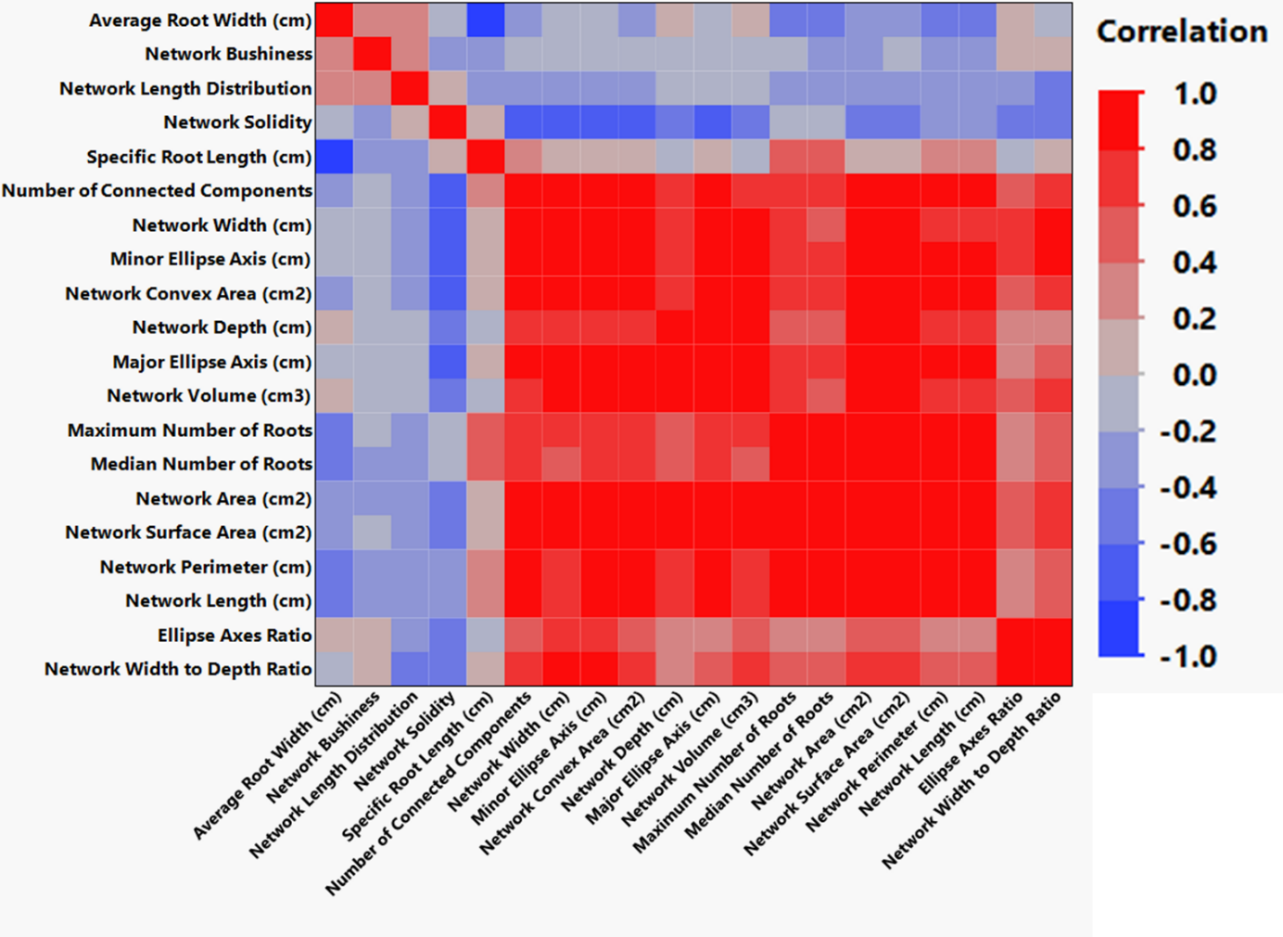
Clustered correlations (Pearson) for all root architecture variables were developed by automated analysis of high-resolution images using GiA Roots software.

We monitored new root growth under different pH conditions (**Figure 7**; **Supplementary Figure S5**), which allowed us to discover the innate (genotype-dependent) plasticity of apple root systems under these conditions. Growth was translated into numerical variables, designated by the automated root image software used for this analysis. The output from the multivariate correlation analysis (**Figure 8**) showed a high level of significant positive correlation (*r >*0.6) among 15 of 20 descriptors including Network Depth, Major Ellipse Axis, Minor Ellipse Axis, Network Area, Network Surface Area, Network Volume, Network Convex Area, Network Perimeter, and Network Length. Additionally, high positive correlations were found among the Maximum Number of Roots, Median Number of Roots, Network Perimeter, and Network Length. Specific Root Length was highly negatively correlated with Average Root Width. Mixed model analysis for all root architecture variables showed that pH displayed the most significant (*p* ≥0.05) effects on 19 out of 20 root architecture variables (**Table 1**) and less significant effects on the rootstock genotype effect (two out of 20 variables) (**Supplementary Table S1**), with no significant interaction (**Supplementary Table S2**). The LS means and standard errors generated by the mixed model analysis for the main effects and interactions are featured in **Table 1**; **Supplementary Tables S1**, **S2** with Tukey HSD designators for the significant effects. Least square means associated with variables that featured a significant pH effect describing the overall amount of growth of the root systems (Number of Connected Components, Network Depth, Major Ellipse Axis, Network Width, Network Area, Network Convex Area, Network Perimeter, Network Volume, Maximum Number of roots, and Network Surface Area) showed higher means for pH 8 when compared to pH 5.5 and 6.5, however the LS Means for Average Root Width, Network Length Distribution and Network Solidity were higher for pH 5.5 descending to the minimum at pH 8. The LS means that featured a significant effect by the rootstock genotype variable showed that G.210 had the highest network width-to-depth ratio, with G.41 and G.214 showing similar ratios and G.890 displaying the lowest ratio with similar results for the Ellipse Axes Ratio. While none of the pH × rootstock genotype interactions were significant in the analysis, they revealed interesting patterns for the Ellipse Axis Ratio (**Figure 4**), Average Root Width (**Figure 5**), and Network Perimeter (**Figure 6**), revealing the possible contribution of individual rootstock genotypes to the significant main effects in this experiment. For example, the Ellipse Axis Ratio showed a more linear pattern with increasing pH in rootstocks G.41 and G.890 compared to G.210 and G.214; in the Average Root Width G.214 displayed a lower value at pH 6.5 compared to the values at 5.5 and 8 whereas G.41 displayed a descending value with increasing pH.

### 3.3 Leaf nutrient analysis

Leaf nutrient concentrations showed no significant differences in P, K, Ca, Mg, S, B, Zn, Cu, or Fe among rootstocks; however, a significant difference was found in Mn concentration among rootstocks (**Supplementary Table S3**). The pH of the solution affects the concentrations of P, Ca, and Mn. Regression analysis showed a quadratic relationship of P, B, and Fe with the solution pH and a linear relationship of Ca, Mg, and Fe with the solution pH. The interaction between rootstock and solution pH was significant for the concentration of Mn (P ≤0.01), but not for other root macro- or micronutrients (**Supplementary Table S3**).

**Figure 9:**
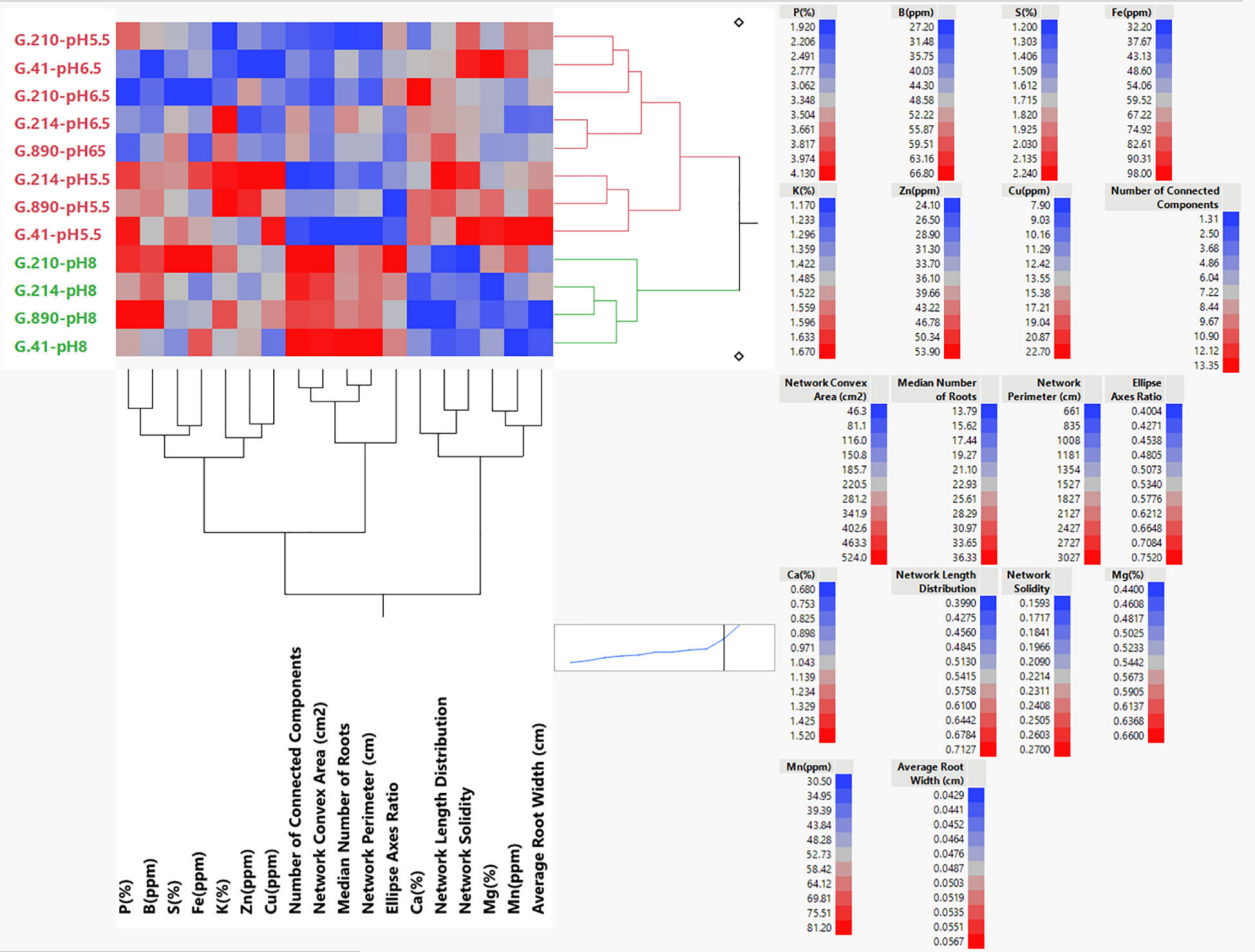
Hierarchical clustering dendrogram using the Ward joining method showing the relationship between pH treatments and genotypic means for root and nutrient parameters. The highest value for each parameter is represented in red, the mid-value in gray, and the lowest value in blue. All the values are shown in **Supplementary Tables S1**, **S3**.

Hierarchical Ward dual cluster analysis (**Figure 9**) resulted in clustering of the genotype × pH interaction, which was mostly due to the pH treatment. There is a distinct cluster for pH 8 and a second cluster that contains pH 5.5 and pH 6.5 with sub clusters that separate the two pH treatments. An exception in this sub-cluster is G.210-pH 5.5, which was similar to the other sub-cluster comprised mostly of rootstock genotypes that had been treated with pH 6.5 solution. Clustering of select architectural variables is related to correlation coefficient among these variables and displayed three distinct groups: the first one comprising of P (%), B (ppm), S (%), Fe (ppm), K (%), Zn (ppm), and Cu (ppm); the second one comprising of Number of Connected Components, Network Convex Area, Median Number of Roots, Network Perimeter Ellipse Axis Ratio; the third cluster containing Ca (%), Network Length Distribution, Network Solidity, Mg (%), Mn (ppm), and Average Root Width. Within these three clusters there were strong similarities between P (%) and B (ppm); S (%) and Fe (ppm); K (%) and Zn (%); Mg (%) and Mn (ppm). These similarities can be better understood in the overall clustered correlation coefficients shown in **Supplementary Figure S4A**, where P, Fe, S, and B are positively correlated to each other and to the Network Convex Area, and negatively correlated with Ca and Network Length Distribution. The loosely correlated group comprising K, Zn, and Cu appeared to be associated with B. A strong correlation between B and P was also observed. Calcium and network length distributions were also strongly associated. The relationships changed when the genotypic correlations were calculated according to the pH level (**Supplementary Figures S4B–D**). The graph featuring pH 5.5 (B) shows four positively correlated groups: (1) P, Network Solidity and Mn; and (2) Ca, Net. Convex Area and Mg; (3) S, Cu, and Fe; (4) K, B, Zn, and Net. Length Distr. The graph featuring pH 6.5 (C) shows more contrasting groups of correlations where positively correlated P, Fe, Mg, and Net. Solidity, Mn and Net. Length Distr. displayed negative correlations with Cu, B, Zn, and Net. Convex Area. Variables K, S, and B were highly correlated. The graph featuring pH 8 (D) shows three highly correlated groups (1) P, K, B, and Cu; (2) Ca, Mg, and Fe; and (3) S, Net. Convex Area, and Mn.

## 4 Discussion

The aeroponic system has proven to be an excellent tool for investigating the root systems of apple rootstocks in controlled environments. In this study, we used an aeroponic system to understand the root dynamics, architecture, and nutrient uptake of apple rootstocks under varying solution pH levels. This method allowed roots to develop in a substrate-free system where all nutrients were supplied in a mist, and roots were able to grow independently of soil interactions. Repeated monitoring and evaluation of the root system as it developed and the responses of roots to solution pH provided data to statistically compare the root architecture and distribution of apple rootstocks for the first time.

To better understand the complexity of the apple root system, multiple root parameters were obtained via image analysis of pictures captured using a specialized high-definition camera. This experiment also utilized computer software available at the time to increase the accuracy of measuring the characteristics of root growth, as well as making it easier and faster. Newer and improved software systems employing Artificial Intelligence are expected to significantly improve the accuracy and types of descriptors for root imaging systems (Goh et al., 2023) capable of monitoring root tip development in real time. This type of development is welcomed in the root research world; however, the evaluation of software properties (positive and negative attributes) is beyond the scope of this study, which utilized the most recommended software, given the type of images that were collected.

The development of lateral roots and associated fine roots is the main component of the root system by which plants explore the soil profile and absorb water and nutrients. The significance of root size and morphology for nutrient uptake has been demonstrated in a number of mathematical models and experiments (Boot, 1990). Relatively thin fine roots, with a specific root length to dry weight ratio, form the smallest parts of the root system. Several Gia Roots descriptors (Number of Connected Components, Maximum Number of Roots, Median Number of Roots) are associated with lateral and fine roots and represent an important root quality that can be clearly monitored and evaluated in an aeroponic system. Small lateral and fine roots are un-suberized and have high permeability compared to older roots. In apple trees, the roots are generally ≤1.0 mm in diameter. In this experiment, we showed that when apple rootstock roots are devoid of media and subjected mostly to gravity, they seem to produce a greater or smaller number of lateral roots according to the pH to which they are subjected. This type of plasticity has never been observed in apple rootstocks and may have implications for how rootstocks perform in different soil pH environments. This is exemplified in **Supplementary Figure S4**, where one can visually see increased root branching at higher pH levels. In other species, phytohormones, such as auxin, seem to promote root development in more alkaline pH environments (Duan et al., 2023), and the regulation of auxin-mediated root cell expansion in *Arabidopsis thaliana* is influenced by apoplastic pH (Barbez et al., 2017). Similar mechanisms may occur in apple roots, where the pH of the treatment solution affects the apoplast pH such that it interacts with hormonal signals. The activity, interplay, and transport of auxins and cytokinins may also be affected by solution pH (Zhang et al., 2023). We did not collect material for the analysis of phytohormones in this experiment but imagine that their levels found in different sections of the root (tips, mid-root, and attachment to the stem) might differ according to pH as well as the expression of genes related to phytohormone pathways. The role of pH in apple seedling development agrees with our observations that lower pH inhibited seedling growth; however, in their study, Nurtaza et al. (2022) used peat substrate with pH adjusted up to neutrality, whereas our study used a pH of 8. In addition to lateral and fine root development, pH affected the overall “lengthening” of the root mass, where pH 8 displayed some of the longest root systems with a significant Ellipse Axis Ratio. This could be because plants at pH 8 grew more, and in the absence of other forces keeping the root system supported, roots just pointed down when they grew over a certain size, which is somewhat supported by the correlation between Network Length and this ratio, but that may not be the only explanation, and more detailed studies are needed to determine if pH affects the gravitropic response of apple rootstock roots.

In this study, the four Geneva^®^ rootstocks shared similar parentage (G.214, G.210, G.890 full sib, and G.41 half sib); therefore, it was not surprising to find similar root behavior among them. However, it is possible that the imaging techniques employed were not fine-tuned to detect smaller phenotypic changes between rootstocks confounded by the larger effects caused by solution pH.

Rootstocks showed variability in their adaptability toaeroponic growth in a nutrient-rich misting system. It was noted that some rootstocks (G.890) showed better and faster root initiation, while other rootstocks (G.214 and G.41) showed 7–9 days of delayed root growth under the aeroponics system.

The results from this study showed that two out of 20 measured root system parameters, the network Width to Depth Ratio (and in a similar way the Ellipse Axis Ratio), were different among the rootstocks where G.890 displayed a significantly downward growing root system compared to G.210. Field observations on G.210 in the NC-140 trial at Traverse City, Michigan USA (Autio et al., 2005) which is a very sandy location showed that several G.210 trees had leaned significantly suggesting a superficial root system (Perry, Ron personal communication) whereas G.890 has been observed to display downward root systems observed on hundreds of thousands micro-propagated plants at the North American Plants (Agromillora Inc.) facility in McMinville, OR, USA (Chang, Yongjian personal communication). G.890, G.214, and G.210 are part of a full sib population that has been used to genetically map dwarfing loci in apple rootstocks as well as other phenotypes, including nutrient uptake (Fazio et al., 2013; Fazio et al., 2014b; Jensen et al., 2014). The ability to select these general architectural trends in aeroponic systems may be useful in genetic mapping with the aid of automated image analysis, instead of using tedious methods that have been used by the breeding program (Fazio et al., 2014a; Fazio et al., 2012b).

Leaf nutrient concentrations did not seem to be significantly affected by rootstock genotype, but this could be explained by the similarity in the selection process of the four Geneva rootstocks, which favored more nutrient-efficient rootstocks, and by the overabundance of nutrients in the misting solution. However, there was an effect of G.41, which seemed to absorb more Mn. The dual clustering analysis revealed that architectural variables describing overall growth (higher with solution pH of 8.0) displayed somewhat higher leaf nutrient concentrations for (P, B, S, Fe, K, Zn, and Cu). Higher levels of Ca and Mg were mostly associated with the root architecture variables of root size and their distribution in the root space (Average Root Width and Network Length Distribution), suggesting a differential ability of nutrient absorption based on size and distribution. Clustered correlation coefficients (**Supplementary Figures S4A–D**) revealed dynamic nutrient content/architecture relationships at different pH levels. Well-known nutrient relationships previously reported on (Fazio et al., 2013; Fazio et al., 2017; Fazio et al., 2020) such as P, K, and B were also observed here; however, the strong positive relationship between K and Ca at pH 5.5 stands out as being very different from all reports where it is usually negative (the higher the potassium, the lower the calcium observed in leaves and fruit), perhaps indicating an effect of acid solution on similar type of absorption between these two essential nutrients at a lower pH. At a higher pH, this relationship normalizes with what has been observed in previous research. Similarly, at lower pH, the usually positive relationship between P, K, and B is not present, suggesting a dynamic for P that changes significantly with pH. A lesser amount of P has been observed in some rootstocks, such as G.41, at lower pH levels (Fazio et al., 2012b), which might explain the relationship change in this dataset as the pH increases. In a previous experiment featuring five pH treatments, 33 rootstocks grafted with Golden Delicious, we noticed that Ca, Zn, Mn, Mo, and to some extent Cu, Fe, and Mg content in leaves, varied significantly with changes in soil pH (Fazio et al., 2012b), which might explain the relationship changes at different pH levels in this study. The relationship between the Network Convex Area (how spread out the roots are in the image), Network Solidity (the density of the root system), and the Network Length distribution and nutrients was selected because of their significant change at different pH levels in the mixed model analysis and their ability to describe the general architecture of the root system. These three parameters also showed changing relationships with different pH levels where Network Solidity displayed a strong correlation at pH 6.5 with Network Length Distribution, which included nutrients such as Mg and Mn where at pH 5.5 and pH 8 the relationship was neutral to negative. The shape of the root system (Network Convex Area) was positively correlated with Ca content in the three separate pH correlation analyses; however, in the whole dataset, it appeared to have a moderate negative relationship (−0.655). Further studies are to understand the complexities of this relationship.

Although genetic factors (quantitative trait loci) have been identified for nutrient absorption and translocation (Fazio et al., 2013), and their performance in long-term field experiments has been verified (Fazio et al., 2020), it is still unclear how these relate to architectural properties (Fazio and Volder, 2009), physiological rootstock effects, such as dwarfing (Fazio et al., 2017), or changes in the biology or composition of the soil (Fazio et al., 2012a). This experiment has begun to reveal these complex relationships, which may have very significant field implications.

Root growth and root architecture are frequently omitted from horticultural research (Wright and Wright, 2004) due to the difficulties in implementing nondestructive root observation tools during plant growth (Silva and Beeson, 2011). The present study was designed to determine the effect of solution pH values (5.5, 6.5, and 8.0) on root dynamics, distribution, development, and architecture of four Geneva apple rootstocks (G.41, G.210, G.214, and G.890) in an aeroponic system for apple trees. We found few significant differences in root architecture parameters among the four Geneva^®^ rootstocks that shared a similar parentage. However, when each rootstock was investigated individually, it showed different root parameters at each solution pH. Additionally, variation between rootstocks was noted in the timing of root initiation and adaptability to aeroponic growing systems.

This is the first report on growing apple rootstocks in aeroponics under varying solution pH, where previous studies of apple trees grown in aeroponic systems were conducted to investigate transformed apples (Zhu and Welander, 1999) and test plant growth regulator translocation (Reed and Buchanan, 1990).

## 5 Conclusion

Solution pH was found to significantly affect most root architecture parameters, suggesting a major effect on apple roots, with implications for soil-type adaptations and irrigation water quality. Since this was the first attempt to investigate the root dynamics and architecture of apple rootstocks using aeroponic systems, there is room for improvement to fully optimize the system to accurately understand apple rootstock, root distribution, and dynamics. Implementing a non-destructive root monitoring system, such as aeroponics, allows for better morphological, physiological and molecular characterization of root systems, linking root parameters to QTL and mineral nutrient uptake traits.

As this study is the first of its kind on apple roots (stocks), we could not compare it to others in the literature. More research is underway in our program, leveraging aeroponic systems that have been upgraded since this experiment to answer questions related to the root architecture, growth dynamics, distribution, and molecular genetics of apple rootstocks. However, it should be pointed out that as the current study was conducted with very young plants, it is necessary to verify the results under similar conditions in the field before final conclusions can be drawn.

## Data availability statement

The original contributions presented in the study are included in the article/**Supplementary Material**. Further inquiries can be directed to the corresponding author.

## Author contributions

AF: Writing – review & editing, Writing – original draft, Methodology, Investigation, Formal analysis, Data curation, Conceptualization. LC: Funding acquisition, Writing – review & editing, Validation, Supervision, Investigation. TR: Writing – review & editing, Writing – original draft, Supervision, Project administration, Methodology, Investigation, Funding acquisition, Conceptualization. GF: Writing – review & editing, Writing – original draft, Visualization, Supervision, Project administration, Methodology, Investigation, Funding acquisition, Formal analysis, Conceptualization.

## Appendix supplementary material: GiA Roots Descriptors

**Average root width**: the mean value of the root width estimation computed for all pixels of the medial axis of the entire root system.

**Ellipse axis ratio**: the ratio of the minor to the major axis of the best fitting ellipse.

**Major ellipse axis**: the length of the major axis of the best fitting ellipse to the network.

**Maximum number of roots**: after sorting the number of roots crossing a horizontal line from smallest to largest, the maximum number is the 84th-percentile value (one standard deviation).

**Median number of roots**: the result of a vertical line sweep in which the number of roots that crossed a horizontal line was estimated, and then the median of all values for the extent of the network was calculated.

**Minor ellipse axis**: the length of the minor axis of the best fitting ellipse to the network.

**Network area**: the number of network pixels in the image.

**Network bushiness**: the ratio of the maximum to the median number of roots.

**Network convex area**: the area of the convex hull that encompasses the image.

**Network depth**: the number of pixels in the vertical direction from the upper-most network pixel to the lower-most network pixel.

**Network length**: the total number of pixels in the network skeleton.

**Network length distribution**: the fraction of network pixels found in the lower 2/3 of the network. The lower 2/3 of the network is defined based on the network depth.

**Network perimeter**: the total number of pixels connected to a background pixel (using an 8-nearest neighbor neighborhood).

**Network solidity**: the total network area divided by the network convex area.

**Network surface area**: the sum of the local surface area at each pixel of the network skeleton, as approximated by a tubular shape whose radius is estimated from the image.

**Network volume**: the sum of the local volume at each pixel of the network skeleton, as approximated by a tubular shape whose radius is estimated from the image.

**Network width**: the number of pixels in the horizontal direction from the left-most network pixel to the right-most network pixel.

**Network width to depth ratio**: the value of the network width divided by the value of network depth.

**Number of connected components**: an integer denoting the number of connected groups of network pixels in the image after image pre-processing. For example, if all network pixels in the thresholded image are connected to all others via a contiguous path of nearest neighbor pixels then the value is 1. If the root network has a break in it somewhere that separates the network into two sub-networks, then the value is 2. Note that a real root should only have 1 connected component, but due to errors in image acquisition and pre-processing, the value may be greater than 1 and can be used as a quality control value.

**Specific root length**: total root length divided by root system volume. Volume is estimated as the sum of cross-sectional areas for all pixels of the medial axis of the root system. The total root length is the number of pixels in the medial axis of the root system.
