# Supplementary material for: Effect of solution pH on root architecture of four apple rootstocks grown in an aeroponics nutrient misting system
Source: Front Plant Sci. 2024 Jun 10;15:1351679. doi: 10.3389/fpls.2024.1351679 (PMC11197432; doi:10.3389/fpls.2024.1351679)
Supplement: Supplementary file 1 [file DataSheet_1.zip › Supplementary Figures.pdf]

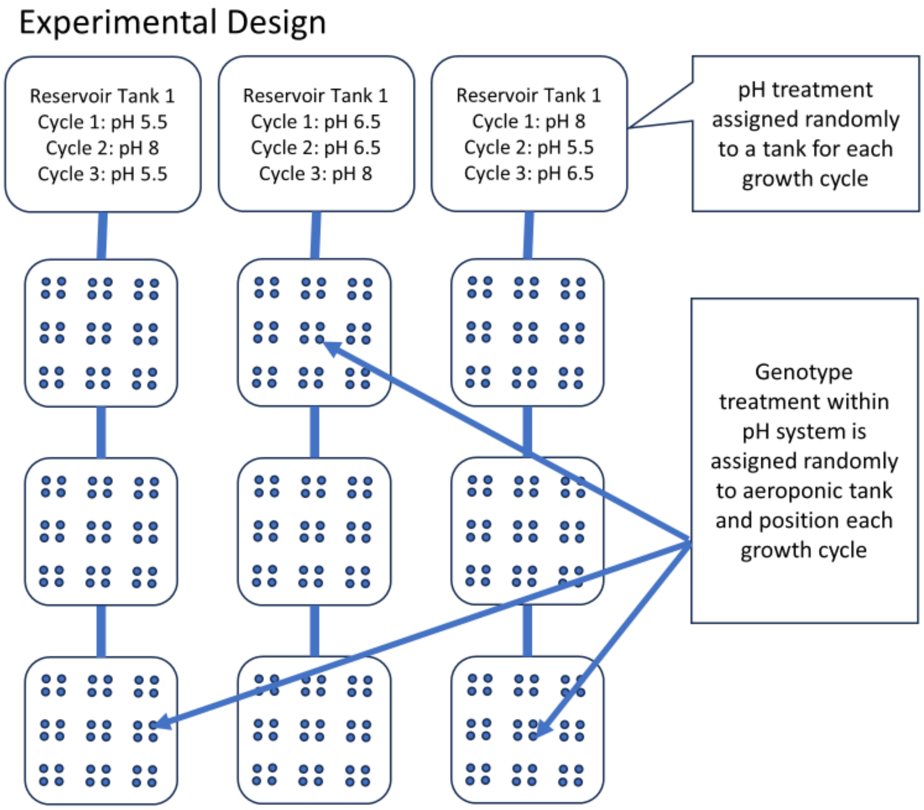

Figure S1. Experimental design showing pH tank systems, position of the plants and randomization for each growth cycle.

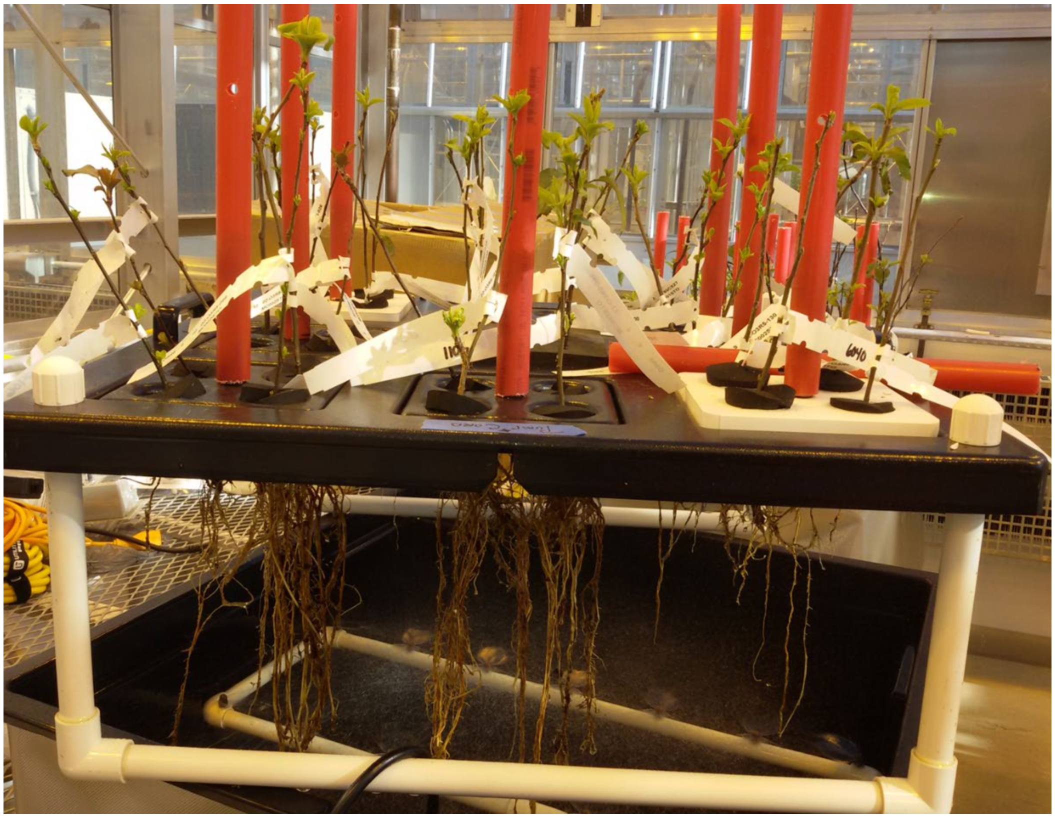

Figure S2. Initial plant setup a few days after transfer into aeroponic systems.

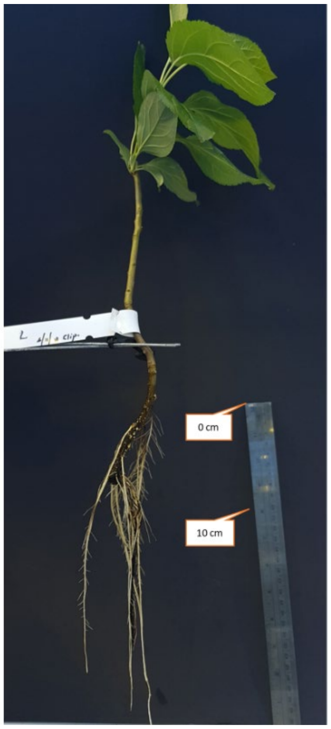

Figure S3. Setup used to collect images with a dimension ruler to ascertain and adjust scale during image processing.

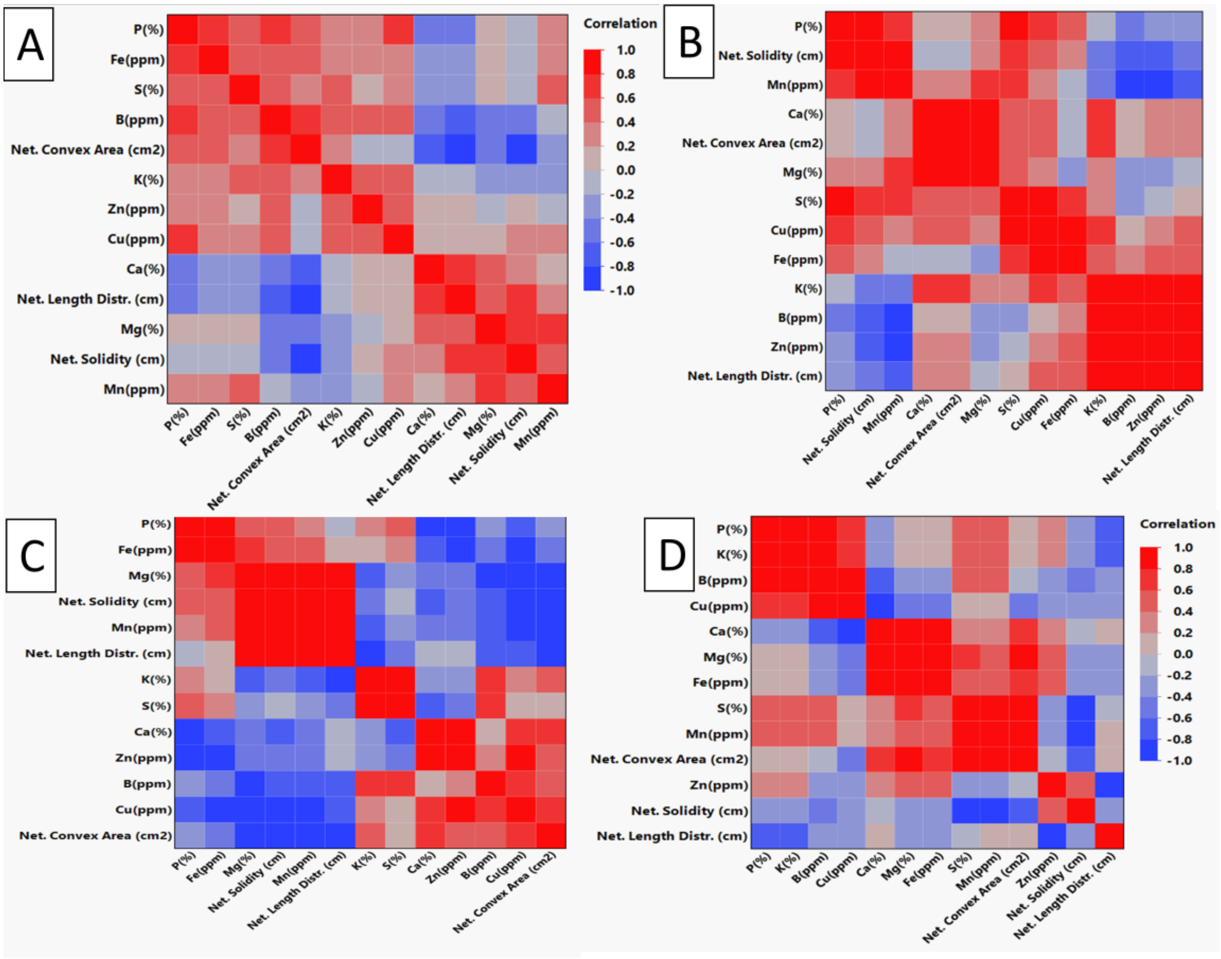

Figure S4. Clustered correlations between genotypic means of nutrients and selected root architecture variables. Panel A represents overall correlations (all pH levels). Panel B, C and D represent pH 5.5, pH 6.5 and pH 8 respectively.

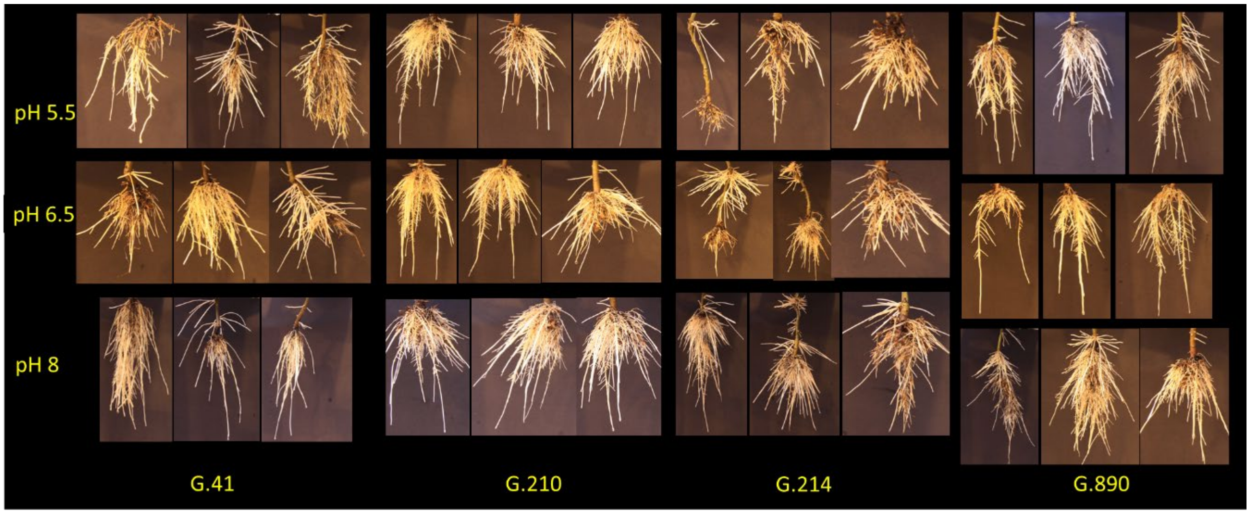

Figure S5. Comparison of sample root systems of plants representing each rootstock (G.41, G.210, G.214, and G.890) grown during cycle 3 in aeroponic solution adjusted to pH 5.5 (top row), 6.5 (middle row) and 8 (bottom row).
