# Supplementary material for: Effect of solution pH on root architecture of four apple rootstocks grown in an aeroponics nutrient misting system
Source: Front Plant Sci. 2024 Jun 10;15:1351679. doi: 10.3389/fpls.2024.1351679 (PMC11197432; doi:10.3389/fpls.2024.1351679)
Supplement: Supplementary file 1 [file DataSheet_1.zip › Supplementary Tables.pdf]

Table S1. Least Square Means and Standard Errors from Mixed Model Analysis (main effects = Rootstock), Tukey HSD and P values for all GiA Roots root architecture variables.

Root Architecture Variable	*G.41*	*Tukey HSD*	*G.210*	*Tukey HSD*	*G.214*	*Tukey HSD*	*G.890*	*Tukey HSD*	*Prob > F*
Average Root Width (cm)	0.049	a	0.049	a	0.048	a	0.047	a	0.7950
Std Error	0.006		0.006		0.006		0.006		
Network Bushiness	1.632	a	1.738	a	1.636	a	1.643	a	0.4189
Std Error	0.100		0.098		0.099		0.095		
Number of Connected Components	6.750	a	6.430	a	7.509	a	8.185	a	0.7545
Std Error	3.598		3.552		3.572		3.510		
Network Depth (cm)	17.416	a	16.453	a	17.879	a	18.932	a	0.3403
Std Error	3.028		2.994		3.009		2.963		
Ellipse Axes Ratio	0.519	b	0.637	a	0.518	b	0.460	b	0.0002
Std Error	0.040		0.038		0.039		0.036		
Network Length Distribution	0.511	a	0.509	a	0.584	a	0.560	a	0.4761
Std Error	0.040		0.034		0.037		0.031		
Major Ellipse Axis (cm)	18.711	a	16.832	a	19.153	a	19.937	a	0.4400
Std Error	4.538		4.493		4.513		4.452		
Maximum Number of Roots	37.402	a	33.977	a	35.721	a	36.921	a	0.9068
Std Error	12.513		12.412		12.455		12.322		
Network Width (cm)	11.755	a	11.925	a	10.969	a	10.863	a	0.9341
Std Error	3.781		3.726		3.749		3.675		
Median Number of Roots	24.188	a	20.012	a	23.722	a	23.792	a	0.5620
Std Error	9.297		9.236		9.262		9.182		
Minor Ellipse Axis (cm)	9.976	a	11.471	a	9.793	a	10.206	a	0.7674
Std Error	3.570		3.527		3.545		3.489		
Network Area (cm2)	33.147	a	29.652	a	32.890	a	34.366	a	0.7961
Std Error	14.859		14.774		14.811		14.698		
Network Convex Area (cm2)	218.414	a	221.380	a	207.984	a	234.333	a	0.9794
Std Error	140.181		138.750		139.364		137.466		
Network Perimeter (cm)	1588.730	a	1259.437	a	1551.256	a	1709.484	a	0.5010
Std Error	876.018		870.402		872.815		865.402		
Network Solidity	0.237	a	0.208	a	0.224	a	0.215	a	0.5069
Std Error	0.016		0.014		0.015		0.013		
Specific Root Length (cm)	449.322	a	450.383	a	484.404	a	515.609	a	0.4901
Std Error	120.576		119.490		119.956		118.517		

Network Surface Area (cm2)	135.897	a	122.403	a	135.312	a	141.829	a	0.8124
Std Error	62.186		61.823		61.979		61.500		
Network Length (cm)	988.670	a	798.632	a	975.954	a	1079.802	a	0.5606
Std Error	569.696		565.907		567.535		562.531		
Network Volume (cm3)	2.300	a	2.282	a	2.271	a	2.321	a	0.9988
Std Error	0.876		0.869		0.872		0.863		
Network Width to Depth Ratio	0.623	ab	0.677	a	0.629	ab	0.509	b	0.0547
Std Error	0.064		0.061		0.062		0.057		

Table S2. Least Square Means and Standard Errors from Mixed Model Analysis (interaction effects = pH x Rootstock) and P values for all GiA Roots root architecture variables.

Rootstock	***G.41***			***G.210***			***G.214***			***G.890***			
pH	***5.5***	***6.5***	***8***	***5.5***	***6.5***	***8***	***5.5***	***6.5***	***8***	***5.5***	***6.5***	***8***	***Prob > F***
Root Architecture Variable													
Average Root Width (cm)	0.056	0.048	0.044	0.051	0.049	0.046	0.0506	0.045	0.049	0.052	0.048	0.042	0.150
Std Error	0.006	0.006	0.006	0.006	0.006	0.006	0.0064	0.006	0.006	0.006	0.006	0.006	
Network Bushiness	1.626	1.603	1.668	1.850	1.756	1.609	1.6736	1.477	1.758	1.636	1.616	1.677	0.284
Std Error	0.131	0.134	0.121	0.124	0.121	0.119	0.1242	0.130	0.121	0.109	0.121	0.118	
Number of Connected Components	2.127	4.777	13.346	2.637	3.329	13.323	1.3145	8.550	12.664	4.985	8.126	11.445	0.552
Std Error	4.153	4.213	3.959	4.015	3.971	3.926	4.0173	4.133	3.963	3.754	3.963	3.918	
Network Depth (cm)	16.522	15.405	20.320	14.577	14.618	20.166	16.0173	18.042	19.577	19.436	17.391	19.970	0.681
Std Error	3.438	3.483	3.293	3.335	3.302	3.269	3.3369	3.423	3.296	3.142	3.296	3.263	
Ellipse Axes Ratio	0.431	0.518	0.609	0.571	0.589	0.752	0.4889	0.468	0.598	0.400	0.451	0.528	0.926
Std Error	0.061	0.0632	0.054	0.056	0.055	0.053	0.0566	0.060	0.054	0.046	0.054	0.053	
Network Length Distribution	0.560	0.549	0.426	0.540	0.575	0.411	0.7127	0.573	0.467	0.644	0.638	0.399	0.831
Std Error	0.077	0.078	0.064	0.067	0.067	0.061	0.0687	0.074	0.065	0.050	0.065	0.062	
Major Ellipse Axis (cm)	14.953	14.822	26.358	12.901	14.166	23.428	14.7567	18.737	23.968	19.303	17.041	23.467	0.606
Std Error	5.089	5.150	4.894	4.951	4.906	4.862	4.9527	5.069	4.898	4.691	4.898	4.854	
Maximum Number of Roots	20.689	34.555	56.961	25.382	33.991	42.556	28.1996	38.150	40.814	32.790	34.430	43.543	0.405
Std Error	13.749	13.888	13.309	13.436	13.336	13.236	13.4407	13.704	13.319	12.853	13.319	13.218	
Network Width (cm)	7.212	7.782	20.271	7.532	7.870	20.374	6.5594	8.527	17.822	8.642	7.670	16.277	0.405
Std Error	4.436	4.507	4.208	4.274	4.222	4.169	4.2766	4.413	4.213	3.966	4.213	4.160	
Median Number of Roots	13.788	22.444	36.332	14.113	19.221	26.701	18.3797	26.732	26.055	22.045	21.824	27.506	0.898

Std Error	10.051	10.136	9.781	9.859	9.798	9.736	9.8617	10.023	9.787	9.503	9.787	9.726	
Minor Ellipse Axis (cm)	5.583	7.408	16.938	7.045	7.928	19.439	5.6891	8.194	15.496	7.994	7.817	14.806	0.357
Std Error	4.084	4.140	3.903	3.956	3.915	3.873	3.9580	4.066	3.907	3.714	3.907	3.866	
Network Area (cm2)	24.277	23.824	51.339	18.907	19.339	50.709	22.8654	30.812	44.993	33.062	27.065	42.972	0.811
Std Error	15.920	16.041	15.540	15.649	15.563	15.477	15.6532	15.882	15.548	15.149	15.548	15.462	
Network Convex Area (cm2)	46.273	95.552	513.417	73.272	66.821	524.047	53.799	129.8949	440.260	156.194	126.368	420.437	0.453
Std Error	157.531	159.454	151.401	153.178	151.784	150.377	153.233	156.9074	151.537	145.003	151.537	150.128	
Network Perimeter (cm)	661.497	1077.487	3027.205	707.859	842.041	2228.411	893.605	1570.8073	2189.355	1502.502	1422.021	2203.929	0.773
Std Error	945.556	953.462	920.694	927.865	922.220	916.552	928.083	943.0381	921.234	895.056	921.234	915.561	
Network Solidity	0.270	0.265	0.176	0.247	0.218	0.159	0.261	0.2263	0.184	0.232	0.225	0.189	0.347
Std Error	0.026	0.027	0.023	0.024	0.023	0.022	0.024	0.0265	0.023	0.019	0.023	0.022	
Specific Root Length (cm)	311.785	450.061	586.119	426.489	458.201	466.458	447.532	543.4934	462.188	431.675	485.404	629.747	0.849
Std Error	133.840	135.322	129.137	130.498	129.429	128.352	130.540	133.3626	129.241	124.246	129.241	128.162	
Network Surface Area (cm2)	98.703	95.170	213.817	76.446	79.976	210.785	93.599	127.1857	185.153	135.491	111.827	178.169	0.283
Std Error	66.698	67.212	65.082	65.547	65.181	64.813	65.561	66.5344	65.117	63.419	65.117	64.748	
Network Length (cm)	382.235	635.131	1948.644	416.021	536.014	1443.862	539.039	1003.3756	1385.448	915.118	912.043	1412.246	0.454
Std Error	616.550	621.869	599.812	604.642	600.841	597.023	604.789	614.8553	600.176	582.537	600.176	596.355	
Network Volume (cm3)	2.303	1.640	2.956	1.500	1.494	3.852	1.755	2.0034	3.054	2.419	1.731	2.814	0.354
Std Error	0.956	0.965	0.928	0.936	0.929	0.923	0.936	0.9539	0.928	0.898	0.928	0.922	
Network Width to Depth Ratio	0.488	0.523	0.859	0.545	0.605	0.882	0.543	0.5280	0.816	0.430	0.434	0.664	0.979
Std Error	0.098	0.101	0.087	0.090	0.088	0.085	0.090	0.0973	0.087	0.075	0.087	0.085	

Table S3. Effect of solution pH on leaf nutrients concentration of four Geneva apple rootstocks grown in aeroponics system 2018.

	pH	P(%)	K(%)	Ca(%)	Mg(%)	S(%)	B(ppm)	Zn(ppm)	Cu(ppm)	Fe(ppm)	Mn(ppm)
Main Effect											
G210	.	3.26	1.33b [z]	0.97	0.54a	0.170a	48.6a	35.5a	11.2a	60.1a	59.4b
G214	.	3.43	1.60a	1.07	0.52.8a	0.177a	50.0a	37.3a	15.4a	61.2a	45.5b
G41	.	3.48	1.40ab	1.08	0.61a	0.165a	41.5a	32.1a	13.9a	67.9a	60.1a
G890	.	3.30	1.61a	1.02	0.52a	0.175a	55.3a	39.2a	14.0a	49.0a	45.1b
Rootstock significance		NS	NS	NS	NS	NS	NS	NS	NS	NS	*
-	5.5	3.82a	1.49a	1.90ab	0.58a	0.180a	51.8ab	42.4a	17.5a	63.6a	62.7a
-	6.5	2.46b	1.47a	1.21a	0.55a	0.162a	36.8b	30.2a	10.0a	43.9a	48.6ab
-	8	3.78a	1.5.3a	0.80b	0.49a	0.175a	58.4a	30.2a	13.4a	68.8a	44.3b
pH significance		*	NS	**	NS	NS	NS	NS	NS	NS	**
Regression		Q**	NS	L*	L*	NS	Q*	NS	NS	Q*	L*

Interaction means											
G210	5.5	3.74	1.17	0.84	0.53	0.168	49.5	31.9	10.2	52.1	63.3abc
	6.5	1.92	1.28	1.52	0.53	0.120	34.6	39.6	11.0	32.2	44.6bcd
	8	3.99	1.57	0.91	0.57	0.224	61.3	35.8	12.8	98.0	69.6ab
G214	5.5	3.92	1.66	1.02	0.53	0.185	54.1	53.9	22.7	85.4	54.7abcd
	6.5	2.86	1.67	1.17	0.53	0.174	37.6	25.8	9.8	51.9	37.6cd
	8	3.50	1.47	0.74	0.44	0.172	58.4	32.1	13.6	46.4	44.1bcd
G41	5.5	4.08	1.45	1.27	0.65	0.193	48.6	32.6	22.2	64.4	81.2a
	6.5	2.75	1.24	1.05	0.66	0.147	27.2	24.1	7.9	54.2	70.7ab
	8	3.46	1.47	0.91	0.52	0.151	45.9	37.9	10.3	82.3	30.5d
G890	5.5	3.53	1.67	1.27	0.60	0.172	54.9	51.1	15.0	52.3	51.5bcd
	6.5	2.29	1.57	1.14	0.51	0.189	44.0	31.6	10.8	38.3	45.8bcd
	8	4.13	1.60	0.68	0.46	0.163	66.8	36.8	16.3	56.8	39.1cd
Interaction significance		NS	NS	NS	NS	NS	NS	NS	NS	NS	**

[z] Mean within columns and section with the same letter are not significantly different using Duncan's at MRT p≤0.05. *,**,*** or NS indicate treatment had a significant effect at P≤0.05 or P≤0.01 or P≤0.001 levels, or had a non-significant effect, respectively.
